# Ibuprofen gargle for chemo- or Chemoradiotherapy-induced Oral Mucositis: a feasibility study

**DOI:** 10.1186/s40780-020-00168-6

**Published:** 2020-06-01

**Authors:** Takeshi Ioroi, Naomi Kiyota, Yoshinori Imamura, Masaaki Tanda, Shiori Aoki, Mamoru Okuno, Kazuhiro Yamamoto, Ryohei Sasaki, Ken-ichi Nibu, Hironobu Minami, Midori Hirai, Ikuko Yano

**Affiliations:** 1 Department of Pharmacy, Kobe University Hospital, 7-5-2 Kusunoki-Cho, Kobe, Hyogo 650-0017 Japan; 2 Department of Medical Oncology and Hematology, Kobe University Graduate School of Medicine, 7-5-1 Kusunoki-Cho, Kobe, Hyogo 650-0017 Japan; 3 Kobe University Hospital Cancer Center, 7-5-2 Kusunoki-Cho, Kobe, Hyogo 650-0017 Japan; 4 Division of Radiation Oncology, Kobe University Graduate School of Medicine, 7-5-1 Kusunoki-Cho, Kobe, Hyogo 650-0017 Japan; 5 Department of Otolaryngology-Head and Neck Surgery, Kobe University Graduate School of Medicine, 7-5-1 Kusunoki-Cho, Kobe, Hyogo 650-0017 Japan

**Keywords:** Ibuprofen, Gargle, Cancer, Oral mucositis, One-arm clinical trial

## Abstract

**Background:**

Oral mucositis frequently occurs in cancer patients treated with chemotherapy and chemoradiotherapy (CRT). This study examined the safety and efficacy of ibuprofen gargle in healthy volunteers and patients with chemotherapy- and concomitant CRT-induced oral mucositis.

**Methods:**

We enrolled healthy volunteers and patients with chemotherapy- and CRT-induced oral mucositis. In cohort I, single and multiple doses of ibuprofen gargle (0.6% or 1.0%) were administered to healthy volunteers on day 1 and days 4–10. In cohort II, multiple doses of ibuprofen gargle (0.6%) were administered to patients with complicated grade 2–3 oral mucositis based on the Common Terminology Criteria for Adverse Events (CTCAE) version 4.0. The primary endpoint of cohort I was the treatment-related adverse events (TRAEs) as defined by CTCAE version 4.0. The primary endpoint of cohort II was the change in the visual analogue scale (VAS) pain score from before to 15 min after gargle use on day 3. The incidence and severity of TRAEs were assessed based on the CTCAE version 4.0 and a subjective rating scale completed by healthy volunteers and patients.

**Results:**

In cohort I, 9 of 10 healthy volunteers were evaluable for safety. All 9 healthy volunteers reported the TRAE of oral irritation with single or multiple use of the gargle. In cohort II, 10 patients were enrolled and evaluable for safety and 7 of 10 patients were evaluable for efficacy. The mean change in the VAS pain score from before to 15 min after using the gargle on day 3 was − 1.28 (95% confidence interval: − 2.06, − 0.51), and all patients experienced some degree of pain relief (range: − 0.2 to − 2.5). All 10 patients reported the TRAE of oral irritation. No other TRAEs of ibuprofen gargle were observed in the healthy volunteers and patients.

**Conclusion:**

Despite oral irritation, the ibuprofen gargle appeared to be safe and effective for the pain related to chemo- or CRT-induced oral mucositis. However, ibuprofen-related oral irritation warrants further formulation improvement.

**Trial registration:**

This study was registered with the University Hospital Medical Information Network Clinical Trials Registry (UMIN000014433).

## Background

Oral mucositis frequently occurs in patients with cancer treated with chemotherapy, chemoradiotherapy (CRT), or haematopoietic stem cell transplantation [1, 2]. The mucositis makes it difficult to chew, maintain oral hygiene, and sustain adequate nutrition, causing impaired quality of life and the potential for stopping treatment [2, 3]. Several topical formulations to relieve mucositis pain have been tried, including opioids [4, 5], non-steroidal anti-inflammatory drugs (NSAIDs) [6, 7], and others [8]. In the clinical practice guidelines for the use of anti-inflammatory agents in the prevention and/or treatment of oral mucositis, the evidence supports the use of benzyldamine mouthwash only is the recommendation and suggestion for the prevention of mucositis associated with radiotherapy and CRT, respectively [2]. No guideline was possible for any other anti-inflammatory agents due to inadequate and/or conflicting evidence. Therefore, additional well-designed research is needed for the treatment of oral mucositis pain in cancer patients.

Developed in the 1960s [9], ibuprofen is a potent inhibitor of prostaglandin synthesis that reduces fever, pain, and inflammation [10]. Since ibuprofen is pharmacodynamically active against both cyclooxygenase (COX)-1 and COX-2, it may have unfavorable effects such as gastrointestinal disorders and kidney dysfunction after the systemic administration. However, several reviews and meta-analyses showed that ibuprofen is effective and the least toxic NSAID in adults and children [11, 12]. It is also approved for over-the-counter sale in many countries. In the oral mucosa, the loss of the permeability barrier leads to rapid diffusion of the drug into tissues as compared to the intact areas of the mucosa [13]. Ibuprofen gargle has a high stability and can be easily manufactured compared with indomethacin spray [14]. Therefore, ibuprofen gargle can be a targeted and efficient drug-delivery system for the site of pain in tissues, producing almost no systemic effects [15].

In this study, we investigated the safety of ibuprofen gargle in healthy volunteers, and the safety and efficacy in patients with chemotherapy- and CRT-induced oral mucositis. The purpose of this study was to explore the feasibility of conducting this research on a larger scale. The data collected in this pilot study is intended for use in power and sample size calculations for a future study.

## Methods

### Healthy volunteers and patients

In cohort I, healthy volunteers aged ≥20 y were enrolled.

In cohort II, patients who met the following criteria were enrolled: (1) age ≥ 20 y, (2) active solid malignancy, (3) being treated with chemotherapy or CRT during hospitalization, and (4) grade 2 or 3 chemotherapy- or CRT-induced oral mucositis as per Common Terminology Criteria for Adverse Events (CTCAE) version 4.0. Exclusion criteria were (1) impaired gastrointestinal function or gastrointestinal disease, (2) concurrent severe or uncontrolled concomitant medical conditions, (3) impaired cardiac function or clinically significant heart disease, (4) aspirin-induced asthma, (5) hypersensitivity to any component of ibuprofen gargle, (6) drug or alcohol dependence, (7) unwillingness or inability to comply with the protocol, (8) current use of aspirin, (9) pregnant or nursing, or (10) current participation in another clinical trial, (11) central nervous system metastases.

### Ibuprofen gargle

The ibuprofen gargle was manufactured at the Department of Pharmacy, Kobe University Hospital. The gargle (100 mL) contained ibuprofen 600 mg (0.6%) or 1000 mg (1.0%), sodium hydroxide, sodium hydrogen carbonate, hydrochloric acid (to regulate pH), glycerin, methylparaben, and propylparaben.

### Treatment protocol

**Fig. 1:**
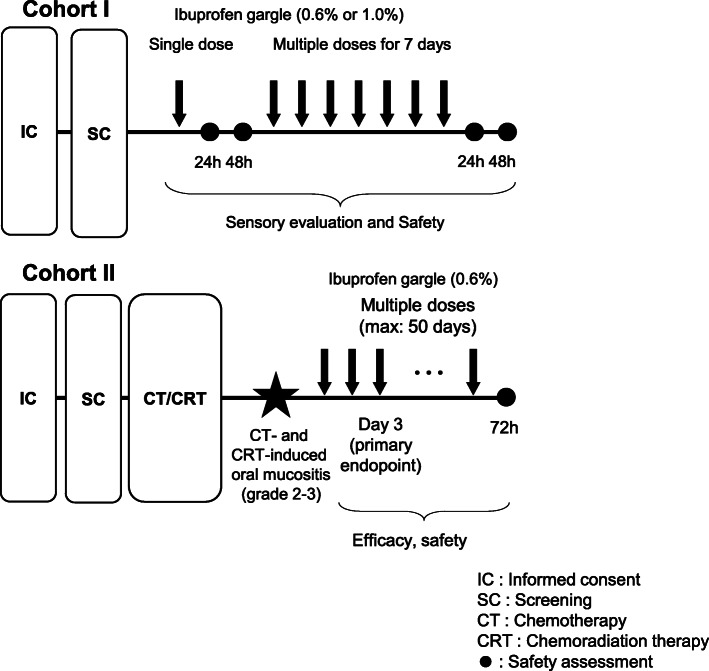
Study design assessing ibuprofen gargle

An open-label, single-arm study was conducted at Kobe University Hospital in Japan. All participants underwent a complete physical examination before enrolment. In cohort I, the healthy volunteers were divided into two groups. Group 1 gargled with 0.6% ibuprofen gargle on day 1 and was checked for treatment-related adverse events (TRAEs) on days 2–3. If there were no serious events, they then used 0.6% ibuprofen gargle on days 4–10 in multiple doses 10 times a day. They were checked for safety on days 11–12. Group 2 followed the same schedule as group 1 but used 1.0% of ibuprofen gargle (Fig. 1).

In cohort II, patients who met the starting criteria were given 0.6% ibuprofen gargle. The starting criteria were defined as haemoglobin ≥8 g/dL, platelet count ≥50,000 cells/μL, aspartate aminotransferase and alanine aminotransferase ≤2.5 times the upper limit of normal (ULN), bilirubin ≤1.5 times the ULN, and serum creatinine < 1.5 times the ULN. All patients were allowed to continue analgesic therapy and basic supportive care for oral mucositis such as dexamethasone oral ointment and/or water-soluble azulene mouthwash. However, a new treatment or dose escalation of basic supportive care for oral pain was prohibited until after the evaluation of primary endpoint on day 3. After the evaluation for the primary endpoint on day 3, patients could continue ibuprofen gargle up to a maximum of 50 days. If pain relief was insufficient and the patient asked for increase in dose, 1.0% ibuprofen gargle was allowed.

The volunteers and patients were given identical instructions to use 10 mL of the gargle for 30–60 s and then expectorate it. The patients were instructed to keep the liquid in contact with the area of mucositis while gargling, and to avoid the simultaneous use of ibuprofen gargle with basic supportive care for oral mucositis. The volunteers and patients were recommended to use ibuprofen gargle before each meal as an example of timing. The use of gargle had to be at least 30 min apart. The gargle could be used one or more times up to a maximum of 10 times a day.

### Endpoints

The primary endpoint of cohort I was TRAEs as defined by CTCAE version 4.0. The primary endpoint of cohort II was the change in the visual analogue scale (VAS) pain score from before to 15 min after using the gargle on day 3. Secondary endpoints in cohort II were the change in the pain score during the entire treatment period and TRAEs.

### Assessment

We required all subjects to record in a diary the number of times they used the ibuprofen gargle each day, the degree of oral irritation (0, none; 1, mild; 2, moderate; 3, severe) with each use, and any other TRAEs. Oral irritation was defined as the tingling sensation other than chemotherapy- and CRT-induced inflammatory in the mouth. Safety was assessed based on the subjects’ diary and the physician consultation to all the healthy volunteers and patients who used the gargle for one or more doses. The period of safety assessment was from the beginning of use until 2 (cohort I) or 3 days (cohort II) after discontinuation of the ibuprofen gargle in accord with CTCAE version 4.0. Patients recorded the intensity of the pain (on VAS) related to their mucositis once daily in the morning before and 15 min after using the gargle. Since the timing of VAS evaluations were variable from 5 to 60 min after the treatment in the previous reports [4–6], we decided the VAS assessment at 15 min after the gargle in order to avoid oral irritation of ibuprofen itself. The data monitoring was performed within the researchers of this study.

### Statistical analysis

Because there were no data available on the efficacy of the ibuprofen gargle, a power calculation was not done for this pilot study. Data on continuous variables were summarized as median and range. Data on safety and efficacy were recorded as mean and 95% confidence intervals (CIs) or the median and range. Categorical variables were summarized by frequencies and percentages. All analyses were performed with R version 3.5.1 (*http://www.r-project.org/*).

## Results

### Participant characteristics

**Fig. 2:**
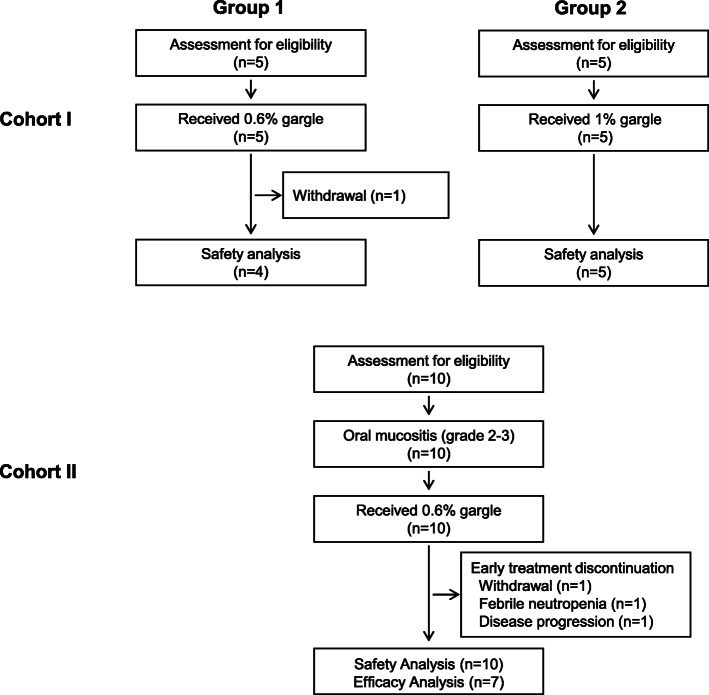
Flowchart of participants through the study

Fig. 2 indicates the flow of participants in each cohort of the study. Ten healthy volunteers were enrolled in cohort I from August to September 2014; 5 in each of the groups described in the Methods. The median age of the volunteers was 24 (range: 23–38) y. Ten patients were enrolled and used one or more doses of ibuprofen gargle in cohort II between October 2014 and November 2015. Because 3 of 10 patients discontinued the treatment before day 3, 10 patients were evaluable for safety and 7 patients were evaluable for efficacy.

Patient characteristics in cohort II are shown in Table 1. On day 1 of using the gargle, the median baseline VAS pain score before use was 3.6 (range: 0.1–7.1).

**Table 1:** Patient characteristics

No.	Age	Sex	Cancer type	TNM stage	Regimen (CT/CRT)	Onset of oral mucositis (Grade 2–3)	Pain VAS (baseline)
1	58	M	Hypopharyngeal carcinoma	T3N2cM0	CDDP+RT	Cycle 2 day 5	6.2
2	73	F	Lacrimal sac tumor	T4N0M0	CDDP+RT	Cycle 2 day 12	0.1
3	77	M	Hypopharyngeal carcinoma	cT4aN2cM0	TPF	Cycle 1 day 13	7.1
4	64	F	Gingival cancer	pT4aN0M0	CDDP+RT	Cycle 2 day 1	4.2
5	75	M	Primary unknown cancer	TxN3M0	CDDP+RT	Cycle 3 day 2	2.0
6	83	M	Oropharyngeal cancer	cT3N2bM0	CDDP+RT	Cycle 1 day 14	4.6
7	32	F	Tongue cancer	pT2cN0M0	CDDP+RT	Cycle 3 day 2	3.2
8	52	M	Maxillary sinus cancer	T4aN0M0	CDDP+ETOP +RT	Cycle 4 day 13	NE
9	25	F	Oral cavity cancer	pT2N1M0	CDDP+RT	Cycle 2 day 8	2.3
10	75	M	Gingival cancer	pT2N2bM0	CDDP+RT	Cycle 2 day 3	3.5

*No*. number, *CT* Chemotherapy, *CRT*: Chemoradiotherapy, *VAS* Visual analogue scale, *M* Male, *F* Female, *CDDP* Cisplatin, *TPF* Docetaxel+cisplatin+fluorouracil, *ETOP* Etoposide, *RT* Radiation therapy, *NE* Not evaluated

### Adherence to treatment

**Fig. 3:**
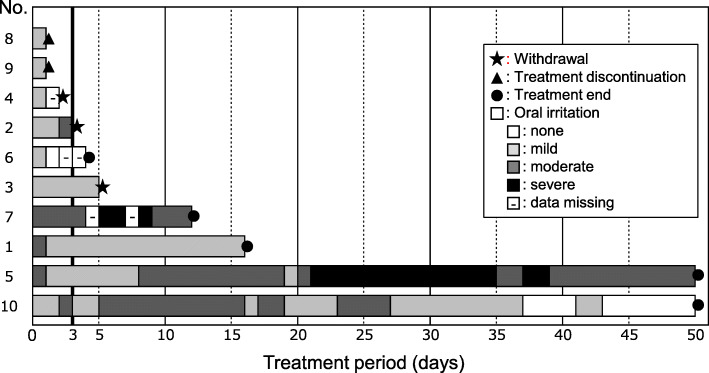
Swimmer plots showing the use of ibuprofen gargle by patients with mucositis, including the degree of oral irritation it caused

One volunteer in group 1 of cohort I withdrew because of non-adherence to the protocol schedule (Fig. 2). Swimmer plots of the patients in cohort II are shown in Fig. 3. In total, 7 of the 10 patients could continue protocol treatment for at least 3 days to assess primary efficacy. The reasons for withdrawal and discontinuation of the protocol treatment within 7 days were grade 3 febrile neutropenia (Patient No. 8), disease progression (No. 9), grade 2 fatigue related to primary disease (No. 4), oral irritation (No. 2), resolution of pain related to CRT-induced mucositis (No. 6), and the ibuprofen gargle was not sufficiently effective (No. 3). The overall median duration of exposure to ibuprofen gargle was 4.5 (range: 1–50) days. None of the patients requested an increase in dose from 0.6 to 1.0% ibuprofen gargle.

### Safety

In cohort I, all 9 healthy volunteers completing the study reported mild or moderate oral irritation with single or multiple uses of the gargle. There were no serious TRAEs or any that resulted in discontinuation of the study drug.

In cohort II, all 10 patients used at least one dose of the ibuprofen gargle, and reported several degrees of oral irritation (Fig. 3). There was no clear relationship between the reported severity of oral irritation and the number of gargles. There were no serious TRAEs. There were no apparent renal impairment induced by ibuprofen gargle among the patients, even if they received cisplatin. As described above, 3 patients had events related to their underlying cancer or its treatment (fatigue, febrile neutropenia, and disease progression) prompting withdrawal from the study before day 3.

### Efficacy

**Fig. 4:**
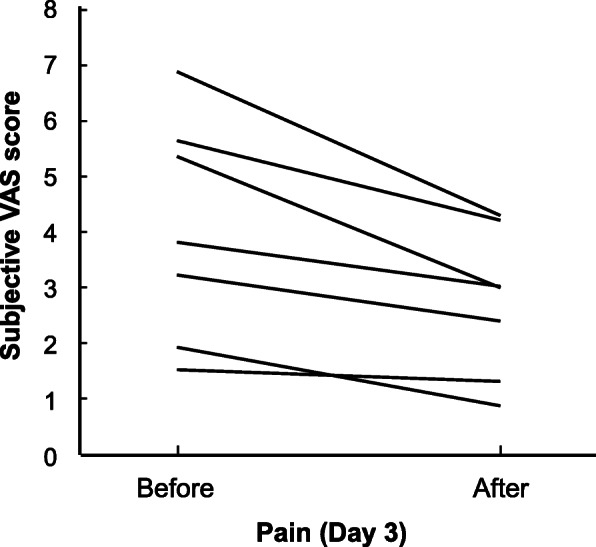
Changes in visual analogue scale (VAS) pain score among patients with oral mucositis on day 3 of treatment with ibuprofen gargle

**Fig. 5:**
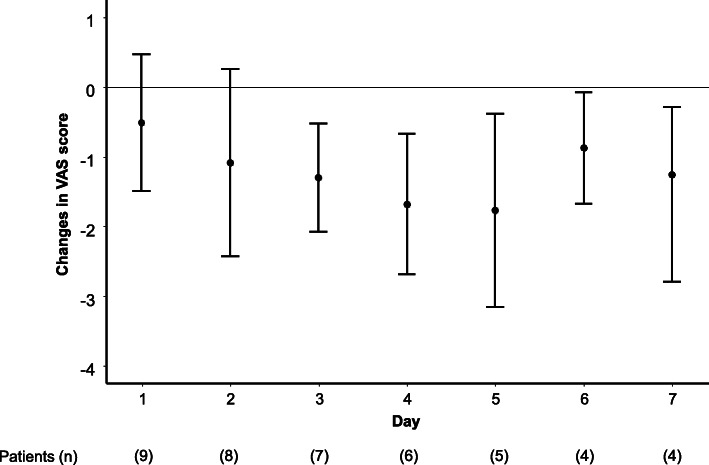
Changes (mean and 95% confidence intervals) in visual analogue scale (VAS) pain score among patients with oral mucositis on days 1–7 of treatment with ibuprofen gargle

The mean values of the VAS pain score before and 15 min after use of the ibuprofen gargle on day 3 were 4.11 (95% CI: 2.29, 5.94) and 2.83 (95% CI: 1.62, 4.04), respectively, and for the mean change of − 1.28 (95% CI: − 2.06, − 0.51) (Fig. 4). All patients experienced some degree of pain relief (median: − 1.0, change range: − 0.2 to − 2.5). The patients used the ibuprofen gargle a median of 4 (range: 1–10) times per day, and the median duration of effect was 20 (range: 10–210) min. The changes in VAS pain scores on days 1–7 for per-protocol patients are shown in Fig. 5. Some degree of pain relief was reported on all 7 days. Among those who continued using it longer (8–50 days), the median change in the pain score was − 1.5 (range: − 0.2 to − 3.0).

## Discussion

There is a pressing need to develop more comfortable and effective treatment to relieve the pain of chemotherapy- and CRT-induced oral mucositis. Our study is the first clinical trial of ibuprofen gargle to treat this painful side effect of cancer treatment. In cohort I, we evaluated the safety of the gargle among healthy volunteers, and in cohort II, we evaluated its efficacy and safety among patients with oral mucositis. Although our data is limited, the ibuprofen gargle did appear to be safe and to afford relief of mucositis-associated pain.

There are several treatment approaches to chemotherapy- and CRT-induced oral mucositis other than gargle or mouthwash [16–18]. But several oral solutions have been evaluated for this purpose. A randomized crossover study reported that the 2% morphine mouthwash for the World Health Organization (WHO) grade ≥ 2 mucositis led to a decrease in the mean VAS pain score on day 3 compared with placebo (a decrease of ~ 2 vs. ~ 1, based on the appearance of the figure shown) [4]. Although the study was incomplete because of the difficulty in finding enough eligible patients, no TRAEs were reported specifically attributable to morphine. A single-arm study of 0.4% ketamine mouthwash to treat WHO grade ≥ 3 mucositis reduced the numeric pain score by 3 points on day 3 compared with baseline [5]. Some patients reported acceptable but metallic taste. In a single-arm study, indomethacin oral spray reduced the 6-grade face scale from 3.6 ± 0.7 to 2.4 ± 0.9 in patients with CTCAE grade 1–3 oral mucositis [6]. No TRAEs were reported specifically attributable to indomethacin in the study. Although our study differs from these studies (in terms of the study drug, the grade of oral mucositis when beginning treatment, and the treatment protocol), all our patients experienced effective pain relief (mean VAS change of − 1.28 with 95% CIs of − 2.06 and − 0.51) on day 3, and reported no TRAEs other than oral irritation. Therefore, ibuprofen gargle can be an effective and safe treatment option for relieving pain related to chemotherapy- and CRT-induced oral mucositis.

In our study, we found no apparent renal impairment induced by ibuprofen gargle among the patients even if who received cisplatin. The only TRAE reported by the participants was oral irritation. When ibuprofen is swallowed in a liquid analgesic formulation, it has been reported to trigger an unpleasant taste (usually described as bitterness) and irritation toward the back of the mouth and throat [19, 20]. In our study, one patient in cohort II withdrawed the ibuprofen gargle because of moderate oral irritation in the mouth or throat. On the other hand, there was no clear relationship between the reported severity of oral irritation and continuation for the two patients who continued using it for 50 days (Fig. 3). Although It might be difficult to completely eliminate the irritation of ibuprofen gargle, the formulation could benefit from changes to reduce the irritation (e.g. adding menthol or peppermint to the gargle as a flavouring agent).

There are two major limitations to this study. First, because of its exploratory nature of this study, the planned sample size was quite small. Therefore, we could not ascertain if the improvement in the VAS pain score was a clinically important difference. Placebo-controlled trials with a larger sample size are needed to thoroughly evaluate the efficacy and safety of ibuprofen gargle in treating the pain of oral mucositis. Second, the only TRAE reported was oral irritation. This may have led to underreporting of other TRAEs associated with ibuprofen gargle because events secondary to the chemotherapy or CRT or the cancer itself may have masked effects induced by the gargle. Therefore, we may have overestimated the safety of ibuprofen gargle. Common ibuprofen TRAEs, including gastrointestinal disorders or abnormal kidney and liver functions, were not observed. We believe at least that the absence of unexpected or severe TRAEs indicates that the ibuprofen gargle treatment was well-tolerated. In fact, in cohort II, 4 of the 10 patients continued using ibuprofen gargle treatment for over 10 days, and two used it for 50 days with no TRAEs other than oral irritation. However, there is some concern that we overestimated the safety of the long-term use because the median duration of exposure to ibuprofen gargle was only 5 days (range: 1–50 days). A longer duration of use should also be addressed in future trials.

## Conclusions

Despite the limitations, ibuprofen gargle appeared in our study to be safe and effective in reducing the pain of chemotherapy- and CRT-induced oral mucositis. However, the oral irritation induced by ibuprofen gargle warrants changes in the formulation to decrease this unpleasant effect.

## Data Availability

The datasets used and/or analyzed during the current study are available from the corresponding author on reasonable request.

## Abbreviations

CI: Confidence interval
CRT: Chemoradiotherapy
CTCAE: Common Terminology Criteria for Adverse Events
NSAIDs: Non-steroidal anti-inflammatory drugs
TRAE: Treatment-related adverse event
ULN: Upper limit of normal
UMIN-CTR: University Hospital Medical Information Network Clinical Trials Registry
VAS: Visual analogue scale
WHO: World Health Organization
